# Supplementary material for: The Complex Emotion Expression Database: A validated stimulus set of trained actors
Source: PLoS One. 2020 Feb 3;15(2):e0228248. doi: 10.1371/journal.pone.0228248 (PMC6996812; doi:10.1371/journal.pone.0228248)
Supplement: S1 Table — This table includes the emotion label answer choices presented to the MTurk raters in the validation task. It lists the four emotion labels presented for each of the 15 types of facial emotion expression. This includes the target label expression, the alternative basic expression(s), and the alternative complex expression(s). (DOCX) [file pone.0228248.s001.docx]

| **Expression** | **Alternative Basic** | **Alternative Complex** |
| --- | --- | --- |
| **Basic Expressions** |  |  |
| Happy | Surprised | Playful  Desirous |
| Sad | Angry | Indifferent  Terrified |
| Fearful | Sad | Hateful  Irritated |
| Disgusted | Sad | Jealous  Panicked |
| Angry | Disgusted | Arrogant  Sarcastic |
| Surprised | Happy | Encouraging  Affectionate |
| **Complex Expressions** |  |  |
| Affectionate* | Surprised | Pensive  Excited |
| Attracted | Happy  Surprised | Relieved |
| Betrayed | Surprised  Disgusted | Embarrassed |
| Brokenhearted | Fearful  Surprised | Flustered |
| Contemptuous | Sad  Disgusted | Worried |
| Desirous* | Surprised | Amused  Excited |
| Flirtatious | Happy  Surprised | Relaxed |
| Jealous | Sad  Fearful | Despondent |
| Lovesick | Fearful  Disgusted | Horrified |

*Raters were shown one alternative basic label and two alternative complex labels, instead of two basic labels because the limited number of positively valanced basic labels.
