# Supplementary material for: The Complex Emotion Expression Database: A validated stimulus set of trained actors
Source: PLoS One. 2020 Feb 3;15(2):e0228248. doi: 10.1371/journal.pone.0228248 (PMC6996812; doi:10.1371/journal.pone.0228248)
Supplement: Attachment — Submitted filename: review of plosone1029.doc [file pone.0228248.s003.doc]

I am very happy to have the opportunity to review this article. Overall, I feel that it is not suitable to publish this paper on our magazine of high level. The main shortcoming is the introduction part. The author simply say that the specific mechanism of research on face recognition is unknown. In fact, there are still many positive results on mechanisms such as Neural pathway, biochemical mechanism etal. At the same time, the author does not explain the complex emotions in a more reasonable way. It is better to have a hypothesis instead of just comparing it with other face databases while this article also missed the introduction of the EKMan expression database which is recognized as a comprehensive expression library.
